# Low-Level Laser Irradiation Exerts Antiaggregative Effect on Human Platelets Independently on the Nitric Oxide Metabolism and Release of Platelet Activation Markers

**DOI:** 10.1155/2017/6201797

**Published:** 2017-12-12

**Authors:** Piotr Rola, Adrian Doroszko, Ewa Szahidewicz-Krupska, Paweł Rola, Piotr Dobrowolski, Robert Skomro, Alicja Szymczyszyn, Grzegorz Mazur, Arkadiusz Derkacz

**Affiliations:** ^1^Wrovasc-Integrated Cardiovascular Centre Provincial Specialist Hospital, Kamienskiego 73a Street, 51-124 Wrocław, Poland; ^2^Department and Clinic of Internal and Occupational Diseases and Hypertension, Borowska 213 Street, 50-556 Wrocław, Poland; ^3^Faculty of Computer Science and Management, Wrocław University of Technology, Wyspiańskiego 27, 50-370 Wrocław, Poland; ^4^Department of Congenital Heart Diseases, Institute of Cardiology, Warsaw, Poland; ^5^Division of Respiratory, Critical Care and Sleep Medicine, Department of Medicine, University of Saskatchewan, Saskatoon, SK, Canada

## Abstract

**Aim:**

The goal of the study is to develop a model allowing to investigate precisely the effect of low-level laser therapy (LLLT) on platelet aggregation and to verify the hypothesis regarding the role of the nitric oxide (NO) bioavailability and platelet activation markers in modulating platelet aggregation.

**Methods:**

A total of 41 healthy volunteers at the age of 21–45 years were investigated. At first, platelet aggregation in response to three agonists (TRAP, ADP, and collagen) was evaluated following previous exposure to different doses of laser radiation (*λ* = 662 nm) to assess the dose-response effect. Subsequently, plasma levels of platelet activation markers (PF4—platelet factor-4 and sP-selectin) as well as the substrate for nitric oxide synthase, L-arginine, and its competitive inhibitors (ADMA—asymmetric dimethylarginine and SDMA—symmetric dimethylarginine) were measured.

**Results:**

All doses of laser irradiation significantly reduced the aggregation. However, the most pronounced effect was observed for 19.7 J/cm^2^. No significant differences in the levels of platelet activation markers nor in the nitric-oxide-metabolic-pathway compounds between analyzed groups were noted.

**Conclusions:**

We have demonstrated in the established *in vitro* experimental model that the LLLT in a reproducible manner decreases the whole blood platelet aggregation regardless of the NO bioavailability or changes in the platelet activation markers.

## 1. Introduction

Numerous studies have shown that the low level laser therapy (LLLT) modulates biological processes in human cells. The most important changes in cellular metabolism include increased activation of intracellular enzymes involved in the respiratory chain and increased synthesis of DNA and RNA as well as regulation of apoptosis [1]. As a result, the low-energy laser radiation has found many applications in a routine clinical practice.

Growing body of attention within the last few years has been paid to LLLT as part of cardiovascular therapy. Recently, we have shown that intravascular irradiation with low-energy laser during percutaneous coronary intervention (PCI) decreases the magnitude of restenosis and may modulate the inflammatory process in vascular wall [2, 3]. Although this method has been demonstrated to be a safe therapeutic option, the effect of LLLT on platelet activity remains unclear. The results of studies carried out so far have been inconsistent. Some of them suggest increased platelet activity following exposure to low-energy laser. Hoffman and Monroe showed that LLLT can enhance the platelet activation [4]. On the other hand, Mohan et al. [5] noted decreased platelet responsiveness following the LLLT. Similar results were observed by Eldar et al. [6] and Brill et al. [7].

Several factors are postulated to modify platelet activity and inflammatory response, among which nitric oxide (NO) is one of the best known [2–7]. The low-energy laser irradiation exposure increases the production of NO in some experimental models conducted *in vitro* and *in vivo* [8, 9]. Nevertheless, the exact mechanism of this phenomenon is unknown [8, 10]. Nitric oxide reduces platelet adhesion and aggregation [11].

Hence, we intended to investigate whether NO is a potential transmitter of LLLT modifying platelet activity. In order to explore the impact of LLLT on platelet activation, the plasma levels of the PF4 and sP-selectin were measured in the samples both at baseline and following the laser irradiation.

## 2. Material and Methods

All experiments were conducted and approved in accordance with the guidelines of the local Bioethics Committee and adhered to the principles of the Declaration of Helsinki and Title 45, U.S. Code of Federal Regulations, Part 46, Protection of Human Subjects (revised November 13, 2001, effective December 13, 2001), and all patients enrolled had signed the informed consent to participate in the study.

Only healthy volunteers aged 21 to 45 years were enrolled in the study. The subjects did not use drugs that will potentially affect the obtained results, such as acetylsalicylic acid and other nonsteroidal anti-inflammatory drugs (grace period was 10 days), and hormonal contraception (washout period of 3 months). Patients taking drugs that affect the metabolism of nitric oxide, including phosphodiesterase inhibitors, dietary supplements containing L-arginine, and nitrates, were also excluded from this experiment.

The study was divided into two phases. The first stage aimed at determining the radiation dose causing the most potent biological effect (analysis of the dose-response curve). It was evaluated by changes in the whole blood platelet aggregation induced by selected agonists (thrombin receptor activating peptide (TRAP-test), ADP (ADP-test), and collagen (COL-test)). Five different doses of irradiation were applied. Immediately after donation, the whole blood (500 *μ*l) was moved to special plastic dots with 20 mm diameter (used in everyday practice to perform the blood group tests) and subsequently irradiated using 5 different energy doses. What is important, during radiation exposure, a control (nonirradiated whole blood obtained from the same patient) was also kept in the dots and next aggregation was measured using an impedance aggregometer (Multiplate® analyzer, Dynabyte Medical, Germany)—paired analyses. In the first stage of the study, the investigated group constituted nine subjects (5 male and 4 female at mean age of 28.9 ± 4.7 y)—nine paired analyses.

In the second stage, only the most effective radiation dose was applied. The same agonists were used, and the same conditions of irradiation were applied which was followed by additional biochemical analyses conducted including platelet activation markers and metabolites of nitric oxide metabolic pathway (see below). Afterwards, platelet aggregation was performed under the same conditions as specified above. This part of the study involved 41 participants—20 women and 21 men (at the mean age of 27.5 ± 7.2 y).

### 2.1. Blood Collection

For platelet aggregation testing, the whole blood was collected into the Sarstedt S-Monovette Hirudin tube (number 04.1944.001). Unlike citrate or heparin, hirudin prevents blood clotting by direct thrombin inhibition, enabling thrombocyte function diagnostics in a native condition.

In order to obtain plasma for determination sP-selectin, PF4, L-arginine, ADMA, and SDMA in the second part of the experiment, blood was collected in the Sarstedt S-Monovette (1.6 mg EDTA/ml blood) tube and within 30 minutes after the collection, it was centrifuged at 1000 ×g for 15 min at 4°C and stored at −20°C until analysis.

### 2.2. Laser Source and Its Validation

In this study, a semiconductor laser (Optel®, Poland) was used, where the diode was optically connected (“pigtailed”) with optical fiber. The optical system was placed at a 15 cm distance from the blood samples. The diffusion system located at the distal end of the optic fiber caused scattering of the laser beam to a circle with a diameter of 2 cm. The wavelength of radiation emitted by the device was 662 nm, and the setup irradiance was 0.050 W/cm^2^ for all radiant exposure doses. During the first stage of the experiment, we planned to use five different doses growing in geometric progression, namely, 2 J/cm^2^, 4 J/cm^2^, 8 J/cm^2^, 16 J/cm^2^, and 32 J/cm^2^.

Due to significant methodological differences in studies conducted so far, we decided to validate the experimental model. Before irradiation of blood samples, the validation of exact parameters of laser radiation reaching the test samples was performed. For this purpose, we used a manual meter PMD100D (Thorlabs Ltd., New Jersey, USA). Measurements were conducted at the same place as the blood samples were irradiated. The results showed that the exact measured radiant exposure doses were 2.4 J/cm^2^, 4.9 J/cm^2^, 9.9 J/cm^2^, 19.8 J/cm^2^, and 39.5 J/cm^2^.

An irradiance of laser was also validated and reached 0.053 W/cm^2^. In order to verify the wavelength of the radiation emitted by the semiconductor laser source, we used a Yokogawa-aq-6370c Optical Spectrum Analyzer (Japan). The analysis of wavelength spectrum, which was dedicated for the experiment, confirmed a monochromatic light (with a peak for a wavelength of *λ* = 662.3 nm).

### 2.3. Aggregation

The whole blood aggregation was measured using an impedance aggregometer (Multiplate analyzer, Dynabyte Medical, Germany). During the analysis, the sample-reagent mixture was stirred with a discardable PTFE- (polytetrafluoroethylene-) coated magnetic stirrer (800 U/min). Preheated to 37°C saline (300 *μ*l) was placed into the test cells with the addition of anticoagulated whole blood (300 *μ*l) and stirred at 37°C; the measurements were started by adding 20 *μ*l of the appropriate agonist: thrombin receptor activating peptide (TRAP-test, Cat. number 6675883190, Roche Diagnostics) at a final concentration of 10.5 *μ*M, ADP (ADP-test, Cat. number 6675794190, Roche Diagnostics) at a final concentration of 3.2 *μ*M, and collagen (COL-test, Cat. number 6675832190, Roche Diagnostics) at a final concentration of 1.05 *μ*g/ml. The change in impedance by the adhesion and aggregation of platelets on the electrode wires was continuously detected. The mean values of the two determinations are expressed in arbitrary units (AU).

### 2.4. Platelet Activation Markers

Plasma concentrations of sP-selectin/CD62P at baseline as well as following the LLLT were determined by a sandwich enzyme immunoassay technique, using commercial ELISA kits (Cat: BBE6, R&D Systems Europe Ltd., UK) with a sensitivity of 0.5 ng/ml, according to the manufacturer's instructions. Optical density 450/620 nm was measured with a BioTek Absorbance Microplate Reader with software Gen5. The intra-assay CV was less 6%, and interassay CV was less 10%.

In order to measure the concentration of PF4 in plasma (at baseline as well as following the LLLT), we used the commercial test human PF4 ELISA kits (Cat: ELH-PF4, RayBiotech Inc., Georgia, USA) with a sensitivity of 20 pg/ml, according to the manufacturer's instructions. Optical density 450 nm was measured with a BioTek Absorbance Microplate Reader with software KC4. The intra-assay CV was less 10%, and the interassay CV was less 12%.

### 2.5. The Metabolites of Nitric Oxide Metabolic Pathway

Evaluation of LLLT-dependent changes in the nitric oxide availability was analyzed by assessing the PRMT-L-Arg/ADMA-DDAH axis. Plasma levels of L-arginine (a substrate for NO synthase) and its methyl derivatives (asymmetric and symmetric dimethylarginine—ADMA, SMDA, competitive inhibitors of the nitric oxide synthase) were measured by a high-performance liquid chromatography (HPLC). Plasma samples and standards extracted a solid-phase extraction cartridge with SCX50 columns (Varian Inc., USA). Eluates were derivatized with *o*-phthaldialdehyde (OPA) and separated by isocratic reversed-phase chromatography on a Symmetry C18 column (150 × 4.6 mm, 5 *μ*m particle size; Waters Corp., USA). Potassium phosphate buffer (50 mM, pH 6.6) containing 12% acetonitrile was used as the mobile phase at a flow rate of 1.1 ml/min. Fluorescence detection was performed at the excitation 340 nm and emission 450 nm wavelengths. The test was performed on a computer controlled by Star Chromatography Workstation software (version 6.3); the device was made by Varian (New York, USA).

### 2.6. Thermal Effect

**Figure 1:**
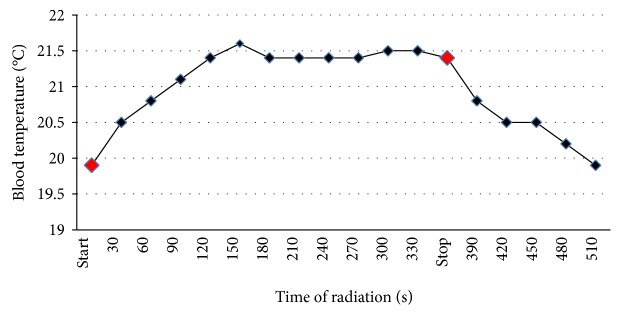
Thermal effect of the blood sample laser irradiation. The maximal increase was observed from 150th second from the beginning of the irradiation procedure (start), and the temperature recovered to the baseline values within the 150 s following cessation of irradiation (stop).

In order to exclude the importance of the possible thermal effect of LLLT on the aggregation results, we decided to measure the temperature increase during irradiation. Measurements were made using an AX5002 AXIOMET Temp Company (Sweden) with measurement accuracy of ±0.1°C. Temperature measurements had been carried out throughout the period of exposure and continued until blood temperature returned to the baseline values (Figure 1).

We observed minimal thermal effect during the laser irradiation. After radiation dose used in the main stage of the study (19.8 J/cm^2^), the absolute increase of blood temperature was 1.5°C from the beginning of the irradiation (start) (Figure 1). It returned to preexposure level after 150 seconds following cessation of irradiation (stop) (Figure 1).

### 2.7. Energy Dose

Based on available literature we selected five different radiant exposure doses to irradiate blood (2.4 J/cm^2^, 4.9 J/cm^2^, 9.9 J/cm^2^, 19.8 J/cm^2^, and 39.5 J/cm^2^). Immediately after irradiation, aggregation tests were carried out.

### 2.8. Statistical Analysis

The data is expressed as mean ± SD. Normality of distribution was verified with Shapiro-Wilk test and the unity of variance by a Levene's test, as appropriate. In the case of quantitative parametric variables, we used Student's *t*-test.

In the case of nonparametric variables, Mann–Whitney's analysis was performed, as appropriate. The analyses were conducted using the Statistica 10.0 (StatSoft) software.

## 3. Results

The first phase of the experiment proved that each of the radiation dose applied caused a significant decrease in platelet aggregation when compared to the nonirradiated control group, regardless of the agonist administered.

**Figure 2:**
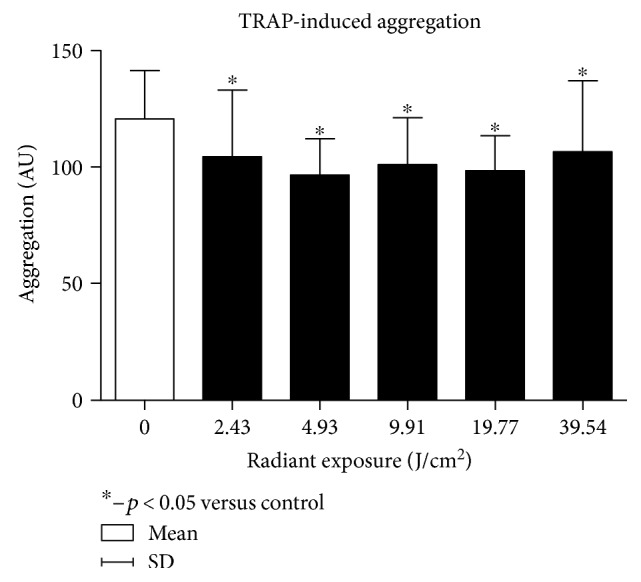
Dose-response effect in the platelet TRAP-induced aggregation.

For TRAP, the greatest decrease was observed for a dose of 4.9 J/cm^2^. Statistically significant but less pronounced decrease in aggregation was also obtained for doses of 2.4 J/cm^2^, 9.9 J/cm^2^, 19.8 J/cm^2^, and 39.5 J/cm^2^ (Figure 2).

**Figure 3:**
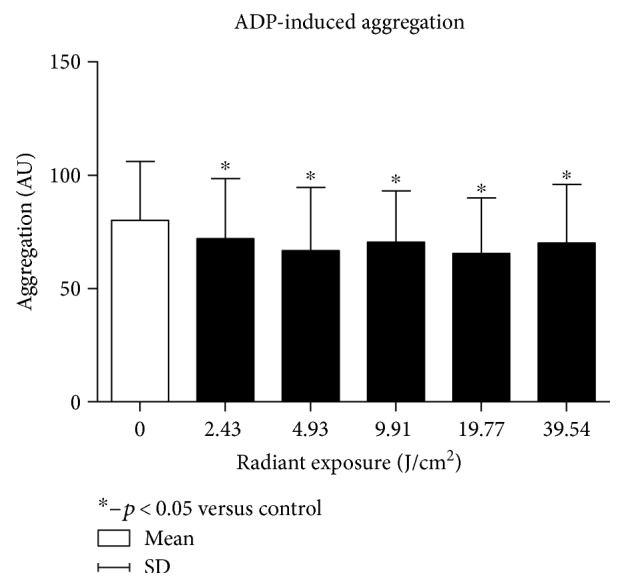
Dose-response effect in the platelet ADP-induced aggregation.

**Figure 4:**
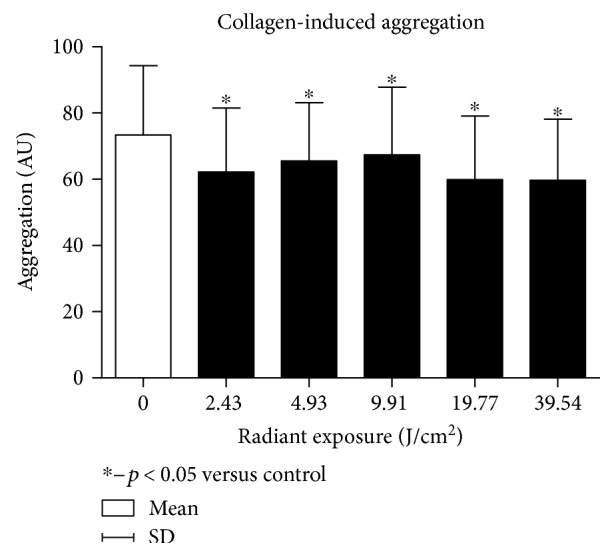
Dose-response effect in the platelet collagen-induced aggregation.

The greatest decrease in aggregation for collagen and ADP was observed for radiant exposure dose of 19.8 J/cm^2^ (*p* = 0.0072 for collagen and *p* = 0.0108 for ADP, resp.) (Figures 3 and 4). No statistically significant differences in aggregation response between the various doses of radiation were observed. Only greater antiaggregatory effect was observed for a dose of 9.9 J/cm^2^ than 39.5 J/cm^2^ for ADP as an agonist. Due to the fact that the greatest biological effect was obtained with a dose of 19.8 J/cm^2^, we used that one in the second phase (Figures 3 and 4).

The second phase of the study involved 41 young healthy participants—20 women and 21 men. For all the agonists (ADP, TRAP, and collagen), the aggregation results following LLLT were statistically significant in comparison to the not irradiated control (not irradiated) sample (Table 1).

In order to verify the involvement of potential molecular mechanisms by which the LLLT affects platelet aggregation, we analyzed changes levels of platelet activation markers as well as the levels of the nitric oxide bioavailability markers (L-arginine—a substrate for the nitric oxide synthase, and its competitive inhibitor—ADMA as well as SDMA characterized by less pronounced inhibitory properties) using energy dose of 19.8 J/cm^2^. There were no statistically significant differences in the concentrations of L-arginine, ADMA, nor SDMA between the two analyzed groups irradiated one and the control without LLLT (Table 1). Similar results were observed regarding plasma platelet activation markers—no statistically significant differences in the levels of PF4 and sP-selectin between irradiated and nonirradiated blood were observed (Table 1).

## 4. Discussion

This is, best to our knowledge, the first human study to investigate the whole blood platelet aggregation following the low-energy laser irradiation in the strictly controlled and reproducible in vitro model. In several previously conducted studies using laser radiation, it is difficult to define clearly the radiation parameters to which the blood had been subjected [4–8]. Most authors suggest that the radiation that emits a laser source reaches directly blood samples without any loss. However, the model has certain limitations especially when semiconductor laser is used, which has already been proved by Hadis et al. [12]. Such simplification makes the results of individual experiments incomparable. In order to avoid the bias, measurements of laser radiation reaching the blood samples were performed. We are aware that the accuracy of measurements may slightly differ from the actual radiation dose. This phenomenon should be related to the difference in radius (10 mm versus 20 mm), the measuring system's manual meter PMD100D and the plastic wells used to store the blood during exposure. However, taking into account the diameter of the scattered laser beam (20 mm), the central position of the blood samples during irradiation, <1 mm thin blood layer, this is the first attempt to describe precisely the experimental model to make it reproducible in subsequent studies. An effective irradiation dose can be assumed to adhere to that one measured by us. The radiation values were different than these ones expected, which had been determined on the basis of theoretical calculations. Due to differences in the radiation doses of LLLT used in studies conducted so far [4–8] and to the absence of evidence for the use of one particular dose, we were the first to perform the initial part of the experiment using such wide-range radiation doses, which subsequently allowed us to select the most effective radiation dose and to illustrate the dose-response effect.

Since the low-energy laser radiation is used in everyday clinical practice [2, 13, 14], we decided to test the whole blood platelet aggregation response. Because the study group was homogeneous, we obtained the model of “physiological” blood response to the LLLT action. Selection of healthy volunteers allowed to limit the number of potential factors affecting the obtained results. In the study, we used the wavelength of *λ* = 662 nm. The available data suggests [15] that two wavelength ranges *λ* = 600–700 nm and *λ* = 800–900 nm are used to modify platelet function. The radiation of these wavelengths is within the “therapeutic window” for LLLT. It is characterized by smaller absorption by water molecules and promotes the action on blood components. The thermal effect of radiation does not appear when the irradiance is below 100 mW/cm^2^ (in our study, the irradiance of 53 mW/cm^2^ was used and the absolute increase of 1.5°C was observed in blood temperature, which recovered to the baseline values within 3 minutes). Considering the fact that the temperature during the analysis in the multiplate aggregometer reaches 37°C, we assume that the mentioned above increase of 1.5°C has a negligible effect on the observed results.

The results of aggregation have shown that low-energy laser radiation reduces platelet aggregation in response at all tested doses of energy and all three agonists used (ADP, TRAP, and collagen). Noteworthy, we are the first to demonstrate that the antiplatelet effect of low-energy laser radiation also applies to the whole blood preparations. The studies published up to date [4–7] were conducted on platelet-rich plasma (PRP). Brill et al. [7] showed reduced activity of platelet aggregation following LLLT (*λ* = 632.8 nm and energy of 0.4–4.2 J) when measured in a classical optical aggregometer in response to TRAP. Interestingly, this effect was dose- and time-elapsing-from-exposure-dependent. Mohan et al. [5] (*λ* = 1060 nm and energy 10–50 J) observed similar results—the authors showed a reduction in aggregation response to collagen, ADP, and ristocetin.

Our data stands in opposition to the study by Hoffman and Monroe [4] where it has been demonstrated that LLLT does not modify the platelet response to thrombin. The authors postulate increased strength of formed clot and increased ability to bind certain clotting factors on the platelet surface after LLLT. It should be noted that PRP and isolated plaques as well as an irradiance dose (12 J/cm^2^, wavelength 650 nm) were used, which could significantly affect the results.

Interestingly, the modulation of aggregation by LLLT is linked between the various agonists. This relationship has been demonstrated between TRAP and ADP. The conducted analyses show that the modification of the whole blood platelet aggregation response to LLLT in case of TRAP was also a predictor of changes in the ADP-induced aggregation, which could result from the cross-talk between the intracellular pathways transducing the signals derived from these two agonists. Present studies and results of this experiment may suggest that modification of the aggregation response after irradiation with a low-energy laser is most likely a consequence of the changes taking place directly in platelets or are due to paracrine effect of nonmorphotic plasma components. Hence, the plasma levels of platelet activation markers before and after irradiation were analyzed. The measurements showed no significant differences between PF4 and sP-selectin levels in both groups. Presented data seems to be incompatible to the one presented by Brill et al. [7], where reduction in P-selectin following LLLT was noted. In our study, we investigated soluble fraction of P-selectin in the plasma and Brill focuses on P-selectin fraction located on the surface of platelets. Taking this into consideration, it can be postulated that LLLT modifies the composition of the platelet membranes but does not affect the factors released to plasma.

Nitric oxide is a potent antiplatelet factor. The low-energy laser radiation modifies cytochrome function, which results in increased nitric oxide production [8, 9]. In our study, there were no significant changes in the levels of L-arginine, ADMA, and SDMA following the LLLT when compared to the baseline values. Hence, this data suggests that LLLT does not change the nitric oxide synthesis. However, taking into consideration these results, we cannot exclude that NO is responsible for the decrease in platelet aggregation. It is possible that direct increase in NO release from blood cells (e.g., from nitroso-hemoglobin (Hb-NO)) [8, 16] is responsible for modification of aggregation. However, in our study, we did not determine the level of this fraction of NO. This would require determination of nitrate or changes in the level of cGMP as a second NO messenger.

## 5. Conclusions

This is the first human study to demonstrate under strictly reproducible conditions that the low-energy laser irradiation decreases the whole blood platelet aggregation in an *in vitro* model on the mechanisms independent on the nitric oxide metabolism and without significant effect on the release of platelet activation markers.

## Figures and Tables

**Table 1:** Comparison of platelet aggregation, nitric oxide bioavailability markers, and platelet activation markers between groups.

	Laser-treated group (after LLLT) *N* = 41 (19.8 J/cm^2^)	Control group (without LLLT) *N* = 41 (0 J/cm^2^)	*p* value
ADP aggregation [AU]	66.8 ± 22.2	72.9 ± 22.7	*p* = 0.0004
TRAP aggregation [AU]	91.5 ± 21.9	105.0 ± 23.5	*p* < 0.0001
Collagen aggregation [AU]	57.7 ± 19.6	64.7 ± 22.3	*p* = 0.0001
L-arginine [*μ*mol/l]	37.6 ± 10.4	38.1 ± 11.5	ns
ADMA [*μ*mol/l]	0.40 ± 0.07	0.42 ± 0.06	ns
SDMA [*μ*mol/l]	0.23 ± 0.08	0.24 ± 0.07	ns
sP-selectin [ng/ml]	26.9 ± 9.8	25.58 ± 9.8	ns
PF4 [ng/ml]	541.9 ± 521.5	519.6 ± 501.2	ns

TRAP: thrombin receptor activating peptide; ADMA: asymmetric dimethylarginine; SDMA: symmetric dimethylarginine; PF4: platelet factor 4.

## Data Access

This study was registered in the EudraCT database. The EudraCT no. 2014-001609-41 has been issued for Protocol code no. POIG.01.01.02-02-001/08project19.
