# Corrigendum to “Low-Level Laser Irradiation Exerts Antiaggregative Effect on Human Platelets Independently on the Nitric Oxide Metabolism and Release of Platelet Activation Markers”

**DOI:** 10.1155/2018/3546870

**Published:** 2018-08-19

**Authors:** Piotr Rola, Adrian Doroszko, Ewa Szahidewicz-Krupska, Paweł Rola, Piotr Dobrowolski, Robert Skomro, Alicja Szymczyszyn, Grzegorz Mazur, Arkadiusz Derkacz

**Affiliations:** ^1^Wrovasc-Integrated Cardiovascular Centre Provincial Specialist Hospital, Kamienskiego 73a Street, 51-124 Wrocław, Poland; ^2^Department and Clinic of Internal and Occupational Diseases and Hypertension, Wroclaw Medical University, Borowska 213 Street, 50-556 Wrocław, Poland; ^3^Faculty of Computer Science and Management, Wrocław University of Technology, Wyspiańskiego 27, 50-370 Wrocław, Poland; ^4^Department of Congenital Heart Diseases, Institute of Cardiology, Warsaw, Poland; ^5^Division of Respiratory, Critical Care and Sleep Medicine, Department of Medicine, University of Saskatchewan, Saskatoon, SK, Canada

In the article titled “Low-Level Laser Irradiation Exerts Antiaggregative Effect on Human Platelets Independently on the Nitric Oxide Metabolism and Release of Platelet Activation Markers” [1], there was an error in the second affiliation where the name of the university was missing. The correct affiliation list is shown above.
